# Texture Feature-Based Machine Learning Classification on MRI Image for Sepsis-Associated Encephalopathy Detection: A Pilot Study

**DOI:** 10.1155/2023/6403556

**Published:** 2023-02-02

**Authors:** Xiao Mo, Xin Xiong, Yijie Wang, Heyi Gu, Yuhang Yang, Jianfeng He

**Affiliations:** ^1^School of Automation and Information Engineering, Kunming University of Science and Technology, Kunming, Yunnan, China; ^2^Department of Critical Care Medicine, The First Affiliated Hospital of Kunming Medical University, Kunming, Yunnan, China

## Abstract

**Objective:**

The objective of this study was to assess the performance of combining MRI-based texture analysis with machine learning for differentiating sepsis-associated encephalopathy (SAE) from sepsis alone.

**Method:**

Sixty-six MRI-T1WI images of an SAE patient and 125 images of patients with sepsis alone were collected. Frontal lobe, brain stem, hippocampus, and amygdala were selected as regions of interest (ROIs). 279 texture features of each ROI were obtained using MaZda software. After the dimension reduction, 30 highly discriminative features of each ROI were adopted to differentiate SAE from sepsis alone using the CatBoost model.

**Results:**

The classification models of frontal, brain stem, hippocampus, and amygdala were constructed. The classification accuracy was above 0.83, and the area under the curve (AUC) exceeded 0.90 in the validation set.

**Conclusion:**

The texture features differed between SAE patients and patients with sepsis alone in different anatomical locations, suggesting that MRI-based texture analysis with machine learning might be helpful in differentiating SAE from sepsis alone.

## 1. Introduction

Sepsis-associated encephalopathy (SAE) is a nonspecific diffuse brain dysfunction caused by sepsis. Clinically, it is mainly manifested as altered mental state and disturbed consciousness without central nervous system (CNS) infection [1]. SAE may happen in more than 50% of septic patients [2, 3]. However, early clinical findings of SAE are usually unspecific, and the diagnosis of SAE is made by evaluating patients' mental status while excluding other diseases caused by brain dysfunction [4]. Because of lack of specific symptoms and effective early diagnostic methods, SAE tends to be missed initially or misdiagnosed, and only 28.1% of SAE patients survive for one year after diagnosis [5, 6]. How to accurately identify the SAE in septic patients has been the focus and difficulty of clinical research. A noninvasive and effective method is therefore needed to help to make precise diagnoses of SAE.

Some studies aimed to identify potential risk factors for SAE based on data collected in routine clinical practice. Zhang et al. [7] indicated that severity of illness, metabolic disturbances, site of infection, and type of microorganism were associated with SAE. Sonneville et al. [8] found that acute renal failure and common metabolic disturbances represent potentially modifiable factors contributing to SAE. Wu et al. [9] investigated the role of dynamic changes of serum levels S100B protein in brain injury and poor outcome of sepsis. Their results showed that elevated serum S100B levels on day 3 were closely associated with SAE. S100B levels on day 3 were diagnostic for SAE with 84.44% specificity, 69.49% sensitivity, and the AUC was 0.819. Zhao et al. [10] proposed a prediction model for SAE using the Medical Information Mart for Intensive Care database, and the results showed that the multivariable logistic regression model only reached an AUC of 0.743. To date, the risk factors associated with SAEare are still under debate, and the results are contradictory. Besides, neuroimaging data was absent in the abovementioned studies.

MRI can reveal the brain injury during SAE [11, 12]. The most consistently reported MRI changes in patients with SAE are interpreted as showing cytotoxic oedema, white matter abnormalities, and ischaemia [13]. However, some studies have found that MRI may be negative in confirmed SAE, the MRI images of SAE patients showed no abnormalities or specificity [14], and diagnostic pattern of imaging findings is unlikely to be observed in patients with SAE [15]. Currently, no consensus on imaging characteristics has been described for SAE diagnosis. This is likely because human eye cannot visualize subtler tissue changes that contains in MRI images. Therefore, more detailed feature extraction and analysis methods for MRI images are definitely required.

Among techniques applied to image analysis, texture analysis is an emergent method that quantifies radiological images and thereby can provide possible imaging biomarkers. By taking advantage of the distributional characteristics of gray levels from medical images, texture analysis could detect subtler image information that cannot be evaluated visually [16]. It has been effectively utilized for type classification and grade discrimination of glioma, identification of small renal tumor types, assessment of prognosis and treatment response in different oncologic disorders, and diagnosis of benign and malignant lung nodules, because these studies are mainly based on statistical analysis and achieved good results [17–21]. Most recently, the rapid development of artificial intelligence provided new idea for texture analysis. By combining machine learning algorithms with texture features, Le et al. [22] efficiently classified transcriptome subtypes in glioblastoma patients. Similarly, Gitto et al. [23] utilized MRI radiomic-based machine-learning method for bone chondrosarcoma classification. Ren et al. [24] predicted the histologic grade of oral squamous cell carcinoma by the same method. These previous studies made a notable progress; however, to the best of our knowledge, texture analysis and machine learning algorithms have not been used as a method to differentiate SAE from sepsis patients.

In this study, we retrospectively collected the 66 and 125 MRI images of SAE and sepsis alone, the texture features of frontal, brainstem, hippocampus, and amygdala were obtained, and CatBoost-based classification models were constructed to differentiate each other. This may help radiologists and clinicians improve the imaging diagnosis accuracy and better understand the differences between SAE and sepsis alone. The rest of this article can be divided into four parts.  Section 2 described work flow of the texture feature-based machine learning method for SAE and non-SAE classification.  Section 3 described the results of the experiment.  Section 4 discussed the results. The conclusion was drawn in Section 5.

## 2. Method

### 2.1. Patients

We retrospectively enrolled 1 patient diagnosed as SAE and 2 patients with sepsis alone between November 2020 and April 2021. All patients had been admitted to the Intensive Care Unit (ICU) of First Affiliated Hospital of Kunming Medical University in Yunnan, China. All the patients aged ≥18 years, and the diagnostic criteria of sepsis were referred to the 2016 Third International Consensus Definition for Sepsis and Septic Shock (Sepsis-3) [25]. The septic diagnostic criteria of Sepsis-3 include the following: (1) infection or a suspected infection; (2) Quick SOFA (qSOFA) ≥ 2, i.e., alteration in mental status, systolic blood pressure ≥ 100 mmHg, or respiratory rate ≥ 22/min; and (3) septic shock can be identified with a clinical construct of sepsis with persisting hypotension requiring vasopressors to maintain MAP ≥ 65 mmHg and having a serum lactate level > 2 mmol/L (18 mg/dL) despite adequate volume resuscitation. Patients with sepsis alone served as a control group.

SAE was defined as a brain dysfunction in the presence of sepsis and without any exclusion criteria, including (1) any preexisting or current CNS disease, current brain disease, concurrent blood disorders, malignancy, cardiac arrest or melanoma, or undergoing cancer chemotherapy, and (2) died within 48 hours after admission [26]. The mental status was assessed using the Confusion Assessment Method for the ICU (CAM-ICU), Glasgow Coma Scale (GCS), and Richmond Agitation-Sedation Scale (RASS). Patients that were CAM-ICU positive were classified as SAE, and patients that were CAM-ICU negative were classified as sepsis alone without brain dysfunction. This study was approved by the Ethics Committee of First Affiliated Hospital of Kunming Medical University, and legal representatives of all patients signed the informed consent before participation.

### 2.2. MRI Data

After evaluating the disease status, all the patients underwent the brain magnetic resonance imaging (MRI) scan under the premise of ensuring safety. MRI was performed using a Siemens MAGNETOM Spectra 3.0 T scanner with head coil. The specific parameters of the MRI scan were as follows: thickness: 6 mm; layer spacing: 10 mm; matrix: 512 × 512; number of excitations: 2; field of view: 19 × 22 cm; and T1WI: repetition time (TR)/echo time (TE) = 500/14 ms. A series of T1WI-MRI images in coronal, sagittal, and transverse positions were obtained. Finally, 66 and 125 MRI images of SAE and sepsis patients were enrolled for further investigation.

### 2.3. Texture Analysis

Texture analysis consists of three main processes, namely, ROI selection, feature extraction, and feature dimension reduction. All these processes were performed in MaZda software (version 4.6, http://www.eletel.p.lodz.Completed in pl/mazda/) [27, 28].

#### 2.3.1. ROI Selection

**Figure 1:**
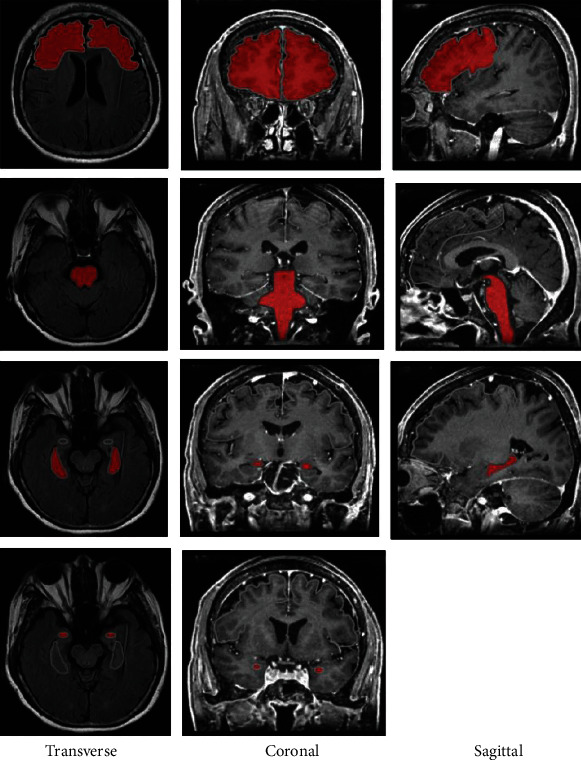
ROIs of the SAE patient (marked red). The first, second, third, and fourth rows were frontal regions lobe, brain stem, hippocampus, and amygdala, respectively. The first, second, and third columns were transverse, coronal, and sagittal images, respectively. The sagittal image of amygdala was absent.

First, the JPG format MRI images were transferred to BMP files and imported into MaZda software. In order to minimize the influence of contrast and brightness variation, normalization was performed to make sure the gray level value range of *μ* ±3*σ* (*μ* and *σ* are the mean gray value and standard deviation, respectively) for each pixel. Then, we selected frontal, brain stem, hippocampus, and amygdala regions as the regions of interest (ROIs). Four ROIs were manually outlined by 2 experienced attending radiologists at the coronal, sagittal, and transverse images of MRI-T1WI images. In SAE patient, we collected 36 frontal lobe images, 16 brainstem images, 10 hippocampal images, and 4 amygdala images; in patients with sepsis alone, we obtained 73 frontal images, 28 brain stem images, 16 hippocampal images, and 8 amygdala images.  Figure 1 shows the ROIs of the SAE patient.

#### 2.3.2. Texture Feature Extraction and Selection

The texture features were extracted using MaZda, an open-source solution for texture analysis. This software is designed primarily for MRI texture analysis and supports a variety of feature selection methods [27]. A major advantage of MaZda lies in its efficiency and ease of use; thus, it has been widely used by researchers worldwide for various texture analysis tasks [28]. But the texture features in MaZda include only one subset recommended by the Image Biomarker Standardization Initiative [29].

Then, 279 features of each ROI were extracted based on image histogram, gray level co-occurrence matrices (GLCM), run-length matrix, absolute gradient, autoregressive model, and wavelets. The detailed definitions of each texture feature computed by Mazda could be found in [27, 28].

Since the dimension of the texture features is higher and not all features will contribute to the classification, a feature selection process is needed to discard redundant texture features. In this study, we used the feature selection method provided by MaZda. This method combined fisher coefficient, mutual information, and average correlation coefficients. Thirty texture features with the highest discriminative power for classification of each ROI were selected for classification.

#### 2.3.3. CatBoost-Based Classification Model Construction and Evaluation

First, we randomly selected 20% of the total samples as the validation set, which did not participate in oversampling and training, and the remaining data was used for constructing the CatBoost-based classification model.

Since the dataset was highly unbalanced, the adaptive synthetic sampling (ADASYN) and random oversampling approach was used to help balance the two classes (SAE and sepsis alone). By prioritizing samples near decision boundaries and focusing on these underrepresented samples, ADASYN can generated new samples from different numbers based on the data distribution [30]. The random oversampling method randomly selects and copies the minority class samples to create additional minority class instances. ADASYN oversampling was used for frontal, brain stem, and hippocampal data, and random oversampling was used for amygdala regions. The processed data was partitioned into a training set and test set, 75% of which were used as training set, and the rest were used as test set.

Then, four CatBoost-based classification models were constructed and trained for each anatomical regions. CatBoost is one of the most recent gradient boosting algorithms over decision trees [31]. It can minimize the problem of overfitting and improve the model of the generalization ability and the robustness, which is particularly suitable for small size of sample and unbalanced data. Some studies found that CatBoost had better performance than traditional algorithms [32, 33].

CatBoost is an improved implementation of gradient enhanced decision tree algorithm, and the classification result is determined by the voting scores of all decision trees. Thus, we tuned the “number of decision trees,” “tree depth,” and “learning rate” to select optimal hyper-parameters.

Number of decision trees is the number of weak learner in each CatBoost model. Using a large number of decision trees could obtain a higher accuracy but also results in long training time. Tree depth indicates a measure of how many splits a tree can make before coming to a prediction, too small value of tree depth might cause difficulties in convergence, and too large value of tree depth might lead to overfitting. Learning rate reflects the magnitude of the parameter update. A small learning rate might lead to gradient vanishing; a large learning rate could cause gradient exposure. Sensible hyperparameters could ensure high classification accuracy, reduce unnecessary computation time, and avoid gradient vanish/explode problem.

In this study, the grid search method was applied to identify the best hyperparameters. Grid search is a common method to select optimal hyperparameters, which sets up the data points in a grid to search for all possible joinpoints, in an aim to find the hyperparameter combination that maximizes the model performance. Because only three parameters need to be optimized for each model, it was computationally possible to perform a grid search through a fixed range of values (number of decision trees = [100, 200, 300, 400, 500, 600, 700, 800, 900, and 1000]; learning rate = [0.001, 0.01, 0.1, 0.2, 0.3, 0.4, 0.5, 0.6, and 0.7]; and tree depth = [1, 2, 3, ⋯, 8, 9, and 10]).

The parameters' range was determined because it can ensure high classification accuracy and reasonable computation time [22]. When the number of decision trees > 1000, learning rate > 0.7, or tree depth > 10, the accuracy did not improve (less than the optimal classification accuracy on test set), and the computation time increased substantially (over twice the average calculation time). When the number of decision trees < 50 and learning rate < 0.001, the accuracy of each model greatly reduced.

The classification accuracy of the test set was used as the evaluation criterion for the model performance. When the test set has the highest classification accuracy, the parameters of the model were taken as the optimal parameters. The generalization ability of each model was evaluated via calculation of classification accuracy and AUC of the validation set.

To provide explanations for the CatBoost classification model, SHapley Additive exPlanations (SHAP) values were obtained to provide a visualization of the contribution of each feature to the classifications. This helps explain the role of each feature for classification in an intuitively understandable way [34, 35].

To determine optimal thresholds for identifying SAE, the features with the highest SHAP values of each anatomical region were selected. Then, these feature values and the corresponding categories were entered into the MedClac (MedCalc Software Ltd., ver. 18.5, Mariakerke, Belgium) software, and the ROC analysis was applied to obtain the best cut-off value for each selected feature.

**Figure 2:**
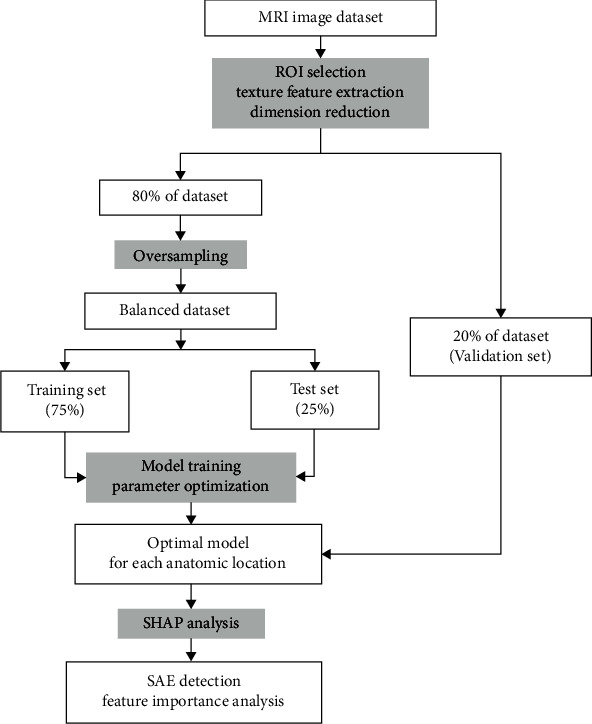
The texture analysis-based model of the classification between SAE and control groups.

CatBoost-based classification models were conducted applying CatBoost and SHAP packages in Python 3.6. All analyses were carried out in Jupyter Notebook environment release 6.0.1. The whole process is shown in Figure 2.

## 3. Results

As shown in Table 1, MaZda software extracted 30 highly discriminative features of frontal lobe, brain stem, hippocampus, and amygdala. Among them, co-occurrence matrix accounted for a large proportion.

### 3.1. Parameter Optimization

We utilized the grid search method for parameter optimization, and Table 2 shows the best parameter combinations which indicated the highest classification accuracy in test set.

### 3.2. Model Performance

As shown in Table 3, the classification accuracy of all models was greater than 80%, and the frontal lobe and brain stem accuracies were slightly reduced in the validation set. Specificity remained constant or elevated, sensitivity was decreased in frontal lobe and brain stem.

**Figure 3:**
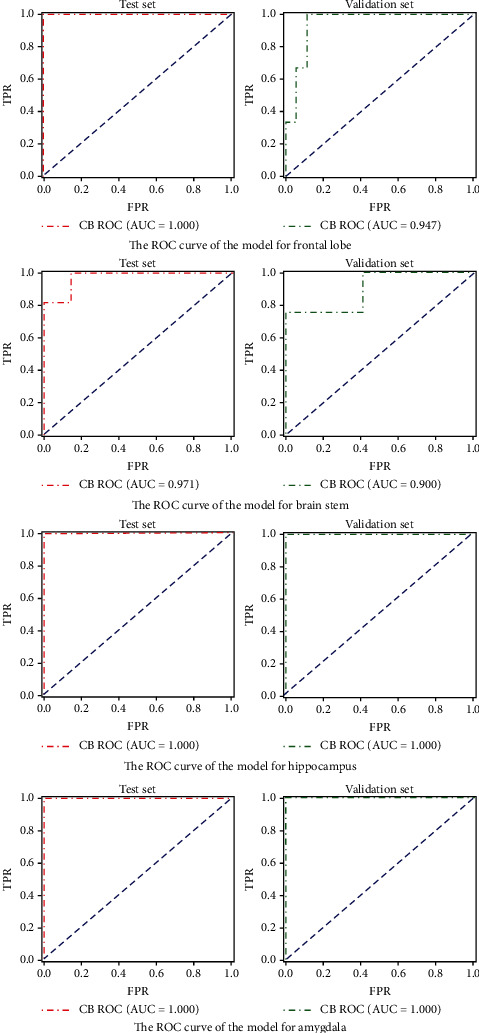
ROC curves of each model in test and validation sets. The first, second, third, and fourth rows were frontal lobe, brain stem, hippocampus, and amygdala, respectively.

Figure 3 shows the receiver operating characteristics (ROC) curves of the model in the test set and validation sets. The AUC of CatBoost was above 0.90 in all four anatomical locations. Among them, AUC, classification accuracy, sensitivity, and specificity of amygdala reached 1.00, and a possible explanation for this could be overfitting caused by the small sample size of amygdala.

### 3.3. Feature Importance Analysis

**Figure 4:**
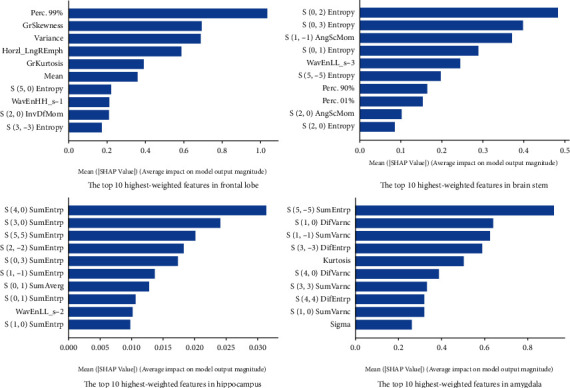
SHAP-based ranking of the features in the each model.

Figure 4 revealed important texture features for each anatomic location, the top 10 highest-weighted features were listed according to the SHAP values. These features varied among anatomic locations. For frontal lobe, the highest-weighted features were Prec. 99%, which is related to histogram; in brainstem, hippocampus, and amygdala, the feature were S(0, 2) entropy, S(4, 0) SumEntrp, and S(5, -5) SumEntrp, which were all related to GLCM. The best cut-off value was obtained using ROC curve analysis in MedCalc software packages. The optimal thresholds for determining the SAE were Perc.99% > 93 for frontal lobe, S(0, 2) entropy > 2.53 for brainstem, S(4, 0) SumEntrp > 1.69 for hippocampus, and S(5, -5) SumEntrp > 1.58 for amygdala.

### 3.4. Comparison with Other Published Data

As shown in Table 4, we compared our results to those of related studies. Zhao et al. [10] constructed a multivariable logistic regression model to identify SAE, and they only reached an AUC of 0.743. Wu et al. [9] found that S100B levels on day 3 were diagnostic for SAE with 84.44% specificity, 69.49% sensitivity, and the AUC was 0.819. In this study, the accuracy of the CatBoost model on each automatic location achieved an accuracy above 0.83 and AUC above 0.90, which was better than the results obtained in the aforementioned studies.

### 3.5. Computation Complexity

In this paper, we evaluate the computational complexity based on execution time. The calculation times of the 4 models were 3.788 s, 2.093 s, 0.707 s and 1.011 s for frontal lobe, brain stem, hippocampus, and amygdala models, respectively. All the computation is performed on a PC with Inter (*R*) Core (TM) i7-1255Uwith32 GB RAM.

## 4. Discussion

The pathogenesis of SAE is not fully understood, and the clinical manifestations is widely variable. At present, there are no well-defined and uniform diagnostic criteria for SAE, and the diagnosis is mainly based on the evaluation of the patients' mental status and the exclusion of other diseases that cause brain dysfunction. Although significant progress has been made in identifying risk factors for SAE, there still remains conflicting evidence in the previous studies [7, 8].

A more effective method is therefore needed to help to make precise diagnoses of SAE. The texture feature-based machine learning method showed good diagnostic performance in preoperative tumor characterization [22–24]. But till now, no study has explored the performance of combining MRI-based texture analysis with machine learning for SAE detection.

In this paper, we applied the texture feature-based machine learning method to construct CatBoost classification models based on different anatomic locations. The results showed that the classification accuracy was above 0.83, and the AUC exceeded 0.90 in the validation set, which is the best result achieved so far in SAE determination. These results suggested that MRI-based texture analysis with machine learning might be helpful in differentiating SAE from sepsis alone.

In order to further explore the feasibility of using texture features and machine learning method for differentiating SAE from sepsis alone, we retrospectively collected the 66 and 125 MRI images of SAE and sepsis alone patients; by extracting the texture features of frontal, brainstem, hippocampus, and amygdala, subtler image information were obtained. Then, CatBoost based classification models for 4 anatomic locations were constructed, respectively. For the validation set, the classification accuracy in each model was above 0.83, and the AUC was above 0.90.

To provide explanations for the CatBoost model, SHAP values were applied to provide a visualization of the contribution of each feature to the classifications. The results show that among the four classification models, the features with the largest SHAP values varied in anatomic locations. For frontal lobe, the highest-weighted features were Prec. 99% indicating the 99th percentile of the grayscale histogram. The value of this feature means that at least 99% of the pixel gray levels are lower than this value. The Perc. 99% of the frontal MRI images of SAE patients was higher than that of the sepsis group, indicating that the greyscale histogram of SAE patients was concentrated rather in the higher gray scale range, while the histogram of the sepsis alone group is distributed in the lower gray scale range, namely, SAE patient showed high signal intensity on T1WI at the frontal lobe area. Some studies have demonstrated SAE accompanied with alterations in permeability of the blood-brain barrier, allowing leakage of low-molecular-weight MRI contrast agents. This caused accumulation of contrast agents in the extravascular intercellular spaces of affected tissues and thus increased signal intensity on T1WI in SAE patients [1, 15, 36]. This may be the reason why the SAE has higher Prec. 99% in frontal lobe than sepsis alone. The highest-weighted features in brainstem, hippocampus, and amygdala were S (0, 2) Entropy, S (4, 0) SumEntrp, and S (5,-5) SumEntrp, respectively. These features were second-order histogram features which described the entropy of the grayscale co-occurrence matrix. These features indicate the randomness of image gray distribution and reflect the spatial distribution characteristics of gray level of texture. In this study, we found that the brain stem, hippocampus, and amygdala in the SAE patient had higher entropy of the grayscale co-occurrence matrix, indicating higher degree of image gray distribution complexity due to the lesions caused by SAE.

There are still several limitations in the study: The sample size was small, and the collected patients were from single center, larger samples (especially an external validation set) that should be used to testify the texture based model in the future work. Only T1WI-MRI images were enrolled in this study, and multimodal MRI data would be collected for further investigation. The link between radiomics and physiopathological features is still unclear, and more in-depth studies are needed in follow-up studies combining the pathophysiological information of SAE patients.

## 5. Conclusion

The present study proposed a combining MRI-based texture analysis with the machine learning method, and this may provide a new perspective to aid in the understanding of the SAE. It could help radiologists and clinicians improve the imaging diagnosis accuracy and better understand the differences between SAE and patients with sepsis alone.

## Figures and Tables

**Table 1:** The 30 highly discriminative features of frontal lobe, brain stem, hippocampus, and amygdala.

Feature types	Frontal lobe	Brain stem	Hippocampus	Amygdala
Histogram-based	Perc. 99%, Perc. 10%, Perc. 01%, mean, Perc. 90%, Perc. 50%, variance	Variance, Perc. 99%, Perc. 90%	Mean, Perc. 10%, Perc. 50%, Perc. 99%, Perc. 01%, Perc. 90%	Perc. 90%, Perc. 99%, kurtosis

Gradient-based	GrKurtosis			GrSkewness
	GrSkewness			

Runlength matrix-based	Horzl_ShrtREmp, Horzl_LngREmph, Horzl_Fraction			

Co-occurrence matrix-derived	S(3, 0) InvDfMom, S(2, -2) entropy, S(3, 0)entropy, S(4, 0) entropy, S(2, 0) entropy, S(4, 0) InvDfMom, S(5,0)entropy, S(2, 0) InvDfMom, S(3, -3) entropy, S(5,0) InvDfMom	S(1, -1) AngScMom, S(0, 2) AngScMom, S(1, 1) AngScMom, S(1, 1) entropy, S(1, -1) entropy, S(1, 0) entropy, S(0, 3) entropy, S(0, 5) entropy, S(0, 1) AngScMom, S(2, 0) entropy, S(0, 1) entropy, S(1, 0) AngScMom, S(0, 1) SumOfSqs, S(0, 5) AngScMom, S(2, 0) AngScMom, S(3, 0) AngScMom, S(0, 2) entropy, S(0, 4) entropy, S(2, -2) entropy, S(3, -3) entropy, S(2, 2) entropy, S(3, 0) entropy, S(4, 0) entropy, S(3, 3) entropy, S(4, -4) entropy, S(4, 4) entropy	S(1, 1) SumEntrp, S(1, 0) SumEntrp, S(2, 0) SumEntrp, S(0, 3) SumEntrp, S(2, -2) SumEntrp, S(0, 3) SumVarnc, S(3, 0) SumEntrp, S(4, 0) SumEntrp, S(4, 4) SumAverg, S(1, -1) SumVarnc, S(5, 5) SumEntrp, S(5, 0) SumAverg, S(5, -5) SumEntrp, S(1, -1) SumEntrp, S(2, 2) SumEntrp, S(0, 1) SumEntrp, S(0, 1) SumAverg, S(1, -1) SumAverg, S(2, 2) SumAverg, S(3, 3) SumEntrp, S(1, 1) SumAveg	S(1, 0) SumVarnc, S(2, 0) SumVarnc, S(1, 0) DifVarnc, S(2, 0) Correlat, S(3, 0) Correlat, S(4, 0) DifVarnc, S(1, 0) Correlat, S(5, -5) SumEntrp, S(3, 0) SumVarnc, S(5, 5) DifEntrp, S(3, 3) SumOfSqs, S(0, 2) SumOfSqs, S(3, -3) DifEntrp, S(3, 0) DifVarnc, S(1, 0) SumOfSqs, S(1, -1) SumVarnc, S(2, 2) SumOfSqs, S(2, 2) DifEntrp, S(0, 4) DifEntrp, S(0 ,5) DifEntrp, S(4, 4) DifEntrp, S(2, 0) DifVarnc, S(0, 3) DifEntrp

Wavelet	WavEnLH_s-2, WavEnLH_s-1, WavEnLL_s-5, WavEnLH_s-4, WavEnLL_s-4, WavEnHH_s-1, WavEnLL_s-3		WavEnLL_s-3, WavEnLL_s-2, WavEnLL_s-1	WavEnHH_s-1

Autoregressive model	Teta2	Teta3		Teta1, sigma

**Table 2:** Parameter optimization for each classification model.

	Frontal lobe	Brain stem	Hippocampus	Amygdala
Number of trees	200	200	100	100
Learning rate	0.5	0.5	0.001	0.001
Depth of trees	5	3	1	1

**Table 3:** Accuracy, sensitivity, specificity, and AUC of models for anatomic locations on the test set and validation set.

	Frontal lobe	Brain stem	Hippocampus	Amygdala
*Test set*				
Accuracy	0.93	0.92	0.83	1.00
Sensitivity	0.88	0.86	0.75	1.00
Specificity	1.00	1.00	0.67	1.00
AUC	1.00	0.97	1.00	1.00

*Validation set*				
Accuracy	0.91	0.89	0.83	1.00
Sensitivity	0.33	0.75	1.00	1.00
Specificity	1.00	1.00	0.80	1.00
AUC	0.95	0.90	1.00	1.00

**Table 4:** Comparison with other published data.

	Method	Accuracy	AUC	Sensitivity	Specificity
Zhao et al. [10]	LASSO + multivariable logistic regression	—	0·743	—	—
Wu et al. [9]	Independent-samples *t*-test/chi-square test	—	0.819	69.49%	84.44%
Our model	CatBoost	0.83-1.00	0.90-1.00	0.33-1.00	0.80-1.00

## Data Availability

The raw data supporting the conclusions of this article will be made available by the authors, without undue reservation.
